# Supplementary material for: The Long Non-Coding RNA HOXA-AS2 Promotes Proliferation of Glioma Stem Cells and Modulates Their Inflammation Pathway Mainly through Post-Transcriptional Regulation
Source: Int J Mol Sci. 2022 Apr 25;23(9):4743. doi: 10.3390/ijms23094743 (PMC9102906; doi:10.3390/ijms23094743)
Supplement: Supplementary file 1 [file ijms-23-04743-s001.zip › supplementary figures.pdf]

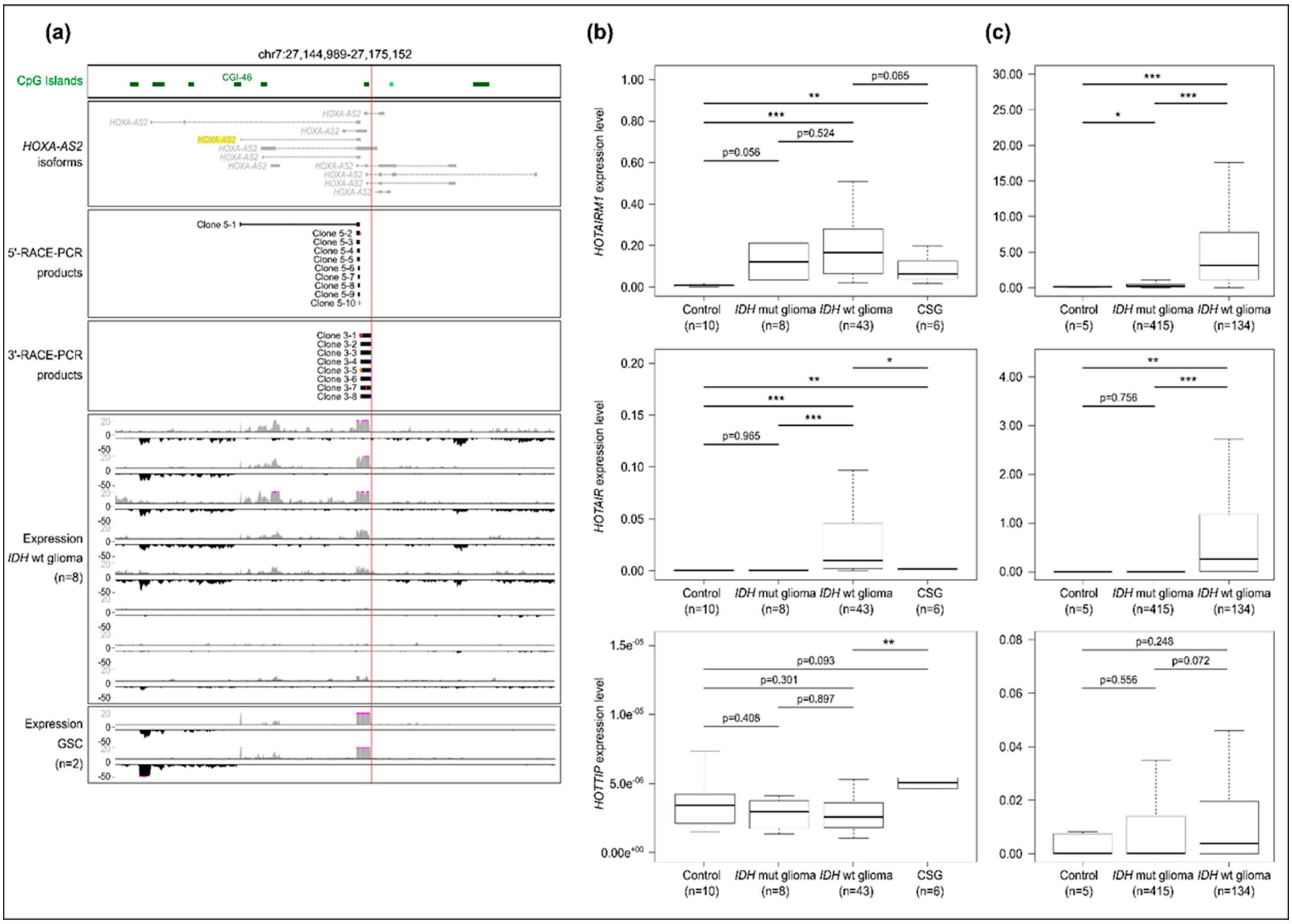

Supp Figure 1: Expression status of *HOTAIRM1, HOTAIR and HOTTIP* in *IDHwt* glioma samples and GSC lines.

(a) Genomic positions of the 5' and 3' RACE-PCR products obtained using an *IDHwt* glioma sample. Strand-oriented RNA-seq signals along the genomic region encompassing *HOXA-AS2* in *IDHwt* (n=8) glioma and Glioma Stem Cell (GSC) (n=2) samples are also shown. **(b) and (c)** Relative expression level of *HOTAIRM1* (upper panel), *HOTAIR* (middle panel) and *HOTTIP* (lower panel) in control, *IDHmut* and *IDHwt* glioma and GSC samples from our cohort analyzed by microfluidic-based RT-qPCR **(b)** and from the TCGA cohort, analyzed by RNA-seq **(c)**. In **(b)** Values are the fold change relative to the geometrical mean of expression of the housekeeping genes *PPIA*, *TBP* and *HPRT1.* *P < 0.05, **P < 0.01, ***P < 0.001 (Mann-Whitney *U*-test).

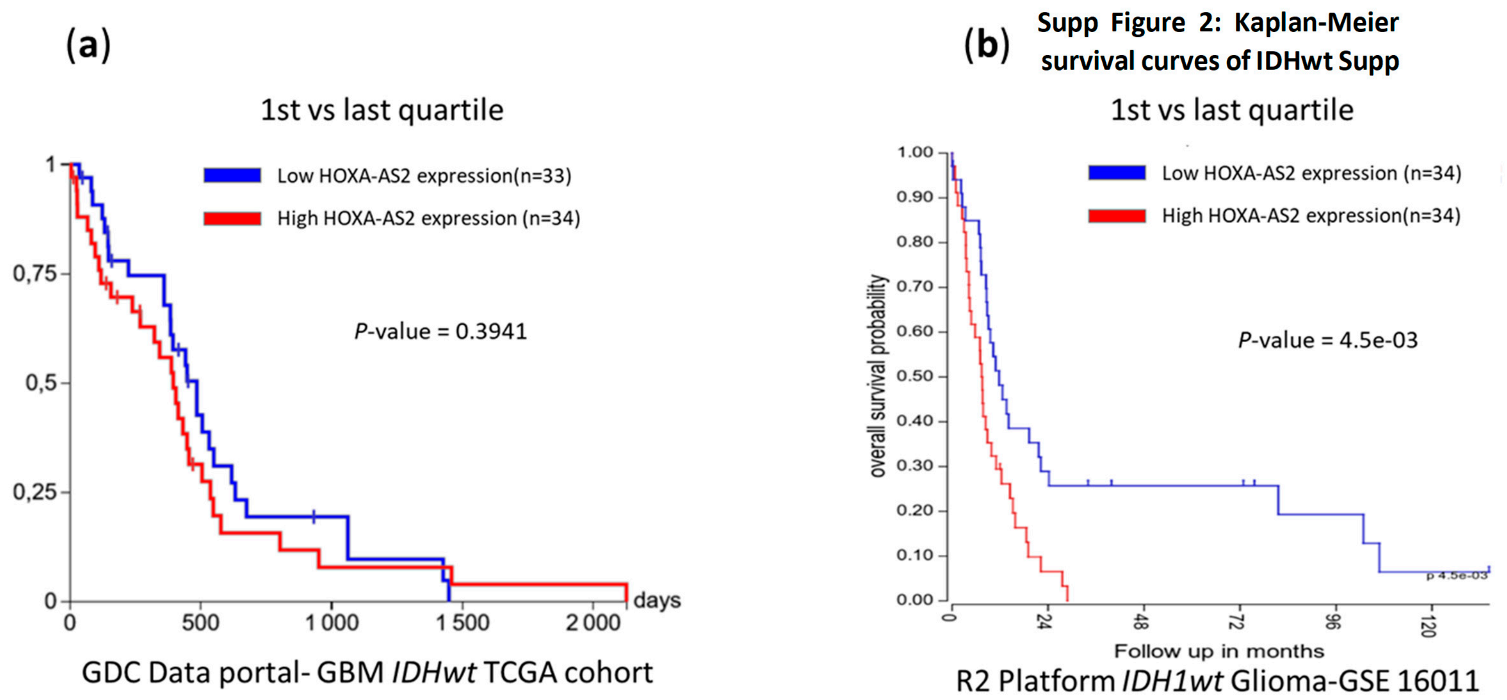

Supp Figure 2: Kaplan-Meier survival curves of IDHwt glioma according to *HOXA-AS2* mRNA expression

(a) Kaplan-Meier survival curves of IDHwt patients of the TCGA cohort from the GDC data portal (https://portal.gdc.cancer.gov/). *HOXA-AS2* expression was assessed by RNA-seq. Comparison is shown for the 1st vs the last expression quartile. (**b**) Kaplan-Meier survival curves of *IDH1w*t patients in GSE16011 (*Gravendeel et al. 2009*) from the R2 Platform (https://r2.amc.nl) of the TCGA cohort from the GDC data portal (https://portal.gdc.cancer.gov/). *HOXA-AS2* expression was assessed with the Affymetrix U133 Plus 2.0 array, probe 230080_at. Comparison is shown for the 1st vs the last expression quartile

Reference :

Gravendeel, L.A.; Kouwenhoven, M.C.; Gevaert, O.; de Rooi, J.J.; Stubbs, A.P.; Duijm, J.E.; Daemen., A.; Bleeker, F.E.; Bralten, L.B.; Kloosterhof, N.K.; De Moor, B.; Eilers., P.H.; van der Spek, P.J.; Kros, J.M.; Sillevis Smitt, P.A.; van den Bent, M.J.; French, P.J. Intrinsic gene expression profiles of gliomas are a better predictor of survival than histology. *Cancer Res.* **2009,** 69, 9065-9072.

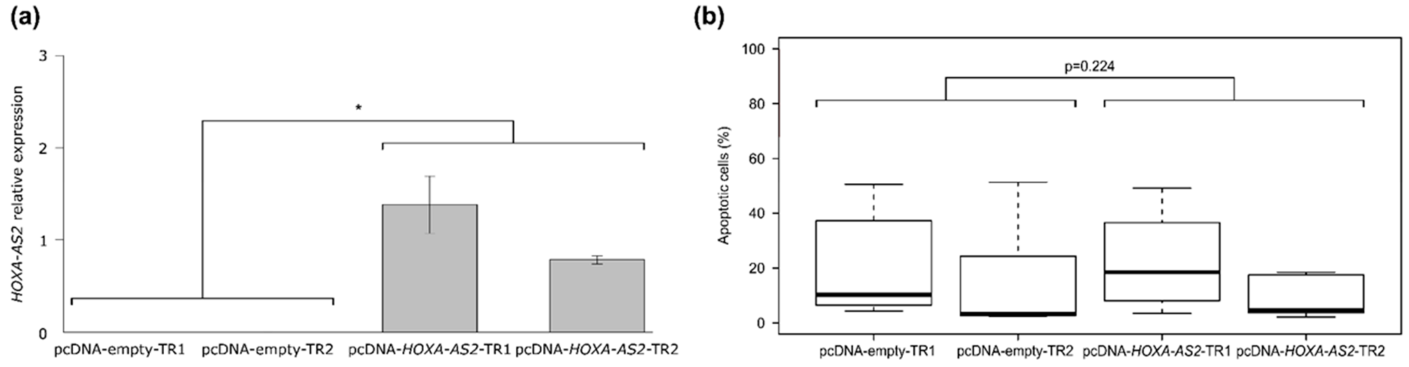

Supp Figure 3: *HOXA-AS2* expression in H9-NSC lines

(a) Relative expression level of *HOXA-AS2* in H9-NSCs transfected with empty vector (n=2) and with a *HOXA-AS2* containing plasmid, analyzed by RT-qPCR. Values are the fold change relative to the geometrical mean of expression of the housekeeping genes *PPIA*, *TBP* and *HPRT1.* **(b)** Apoptosis quantification (n=5) in the two *HOXA-AS2*-expressing H9-NSC lines and their respective H9-NSC control line. TR1 and TR2 refer to the two independent transfection experiments. *P < 0.05 (Mann-Whitney *U*-test).

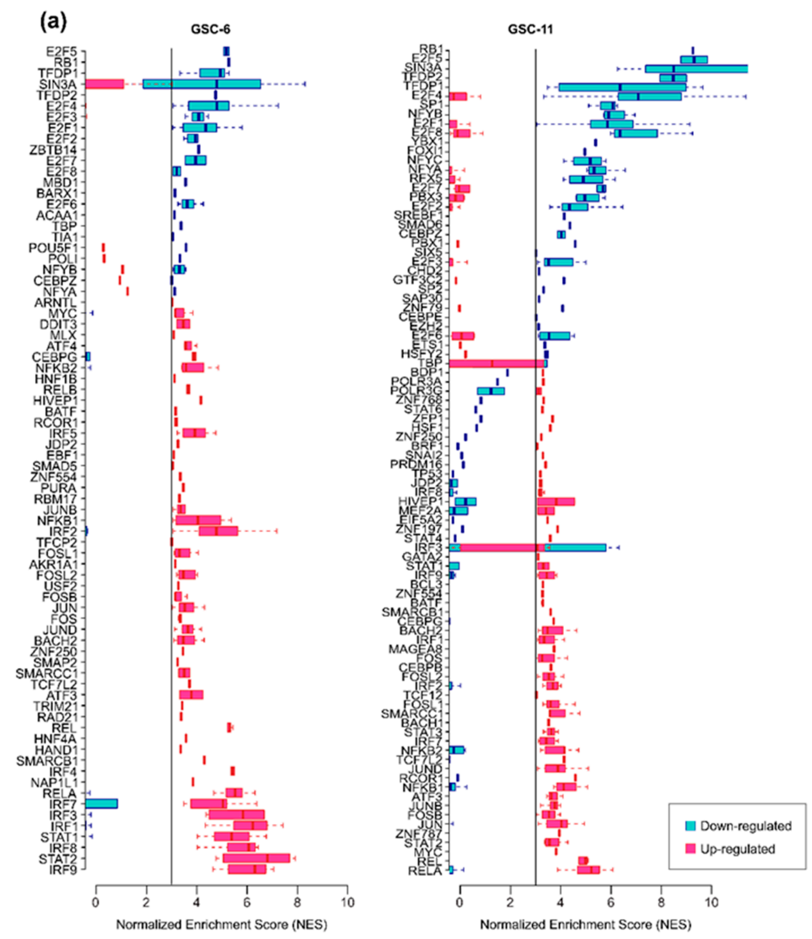

(b)

	R Pearson coeff.	p-value	
E2F8	0.2365	0.0059	**
E2F1	0.2207	0.0104	*
E2F2	0.1645	0.0575	
IRF7	0.1619	0.0617	
IRF9	0.1535	0.0766	
TRIM21	0.1491	0.0854	
NFKB2	0.1432	0.0987	
IRF1	0.0752	0.3876	
STAT2	0.0658	0.4504	
STAT1	0.0430	0.6218	
ATF3	-0.0489	0.5746	
MYC	-0.0519	0.5515	
FOSB	-0.0968	0.2659	

Supp Figure 4: Transcription factor motif enrichment at the promoter of up- and down-regulated genes following *HOXA-AS2* silencing in GSG-6 and GSC-11 cells

(a) Transcription factor motif enrichment at the promoter (defined as the area covering -1 kbp of RefSeq TSS) of up- and down-regulated genes following *HOXA-AS2* silencing in GSG-6 and GSC-11 cells, calculated using *i-cis* Target and represented as normalized enrichment score (NES). When a transcription factor harbors several binding motifs, data are presented as a box plot. **(b)** Correlation between *HOXA-AS2* expression and the transcription factors identified in Figure 5C, established using publicly available RNA-seq data from 134 *IDH*wt samples (TCGA cohort). **P < 0.01, ***P < 0.001 (Mann-Whitney *U*-test).

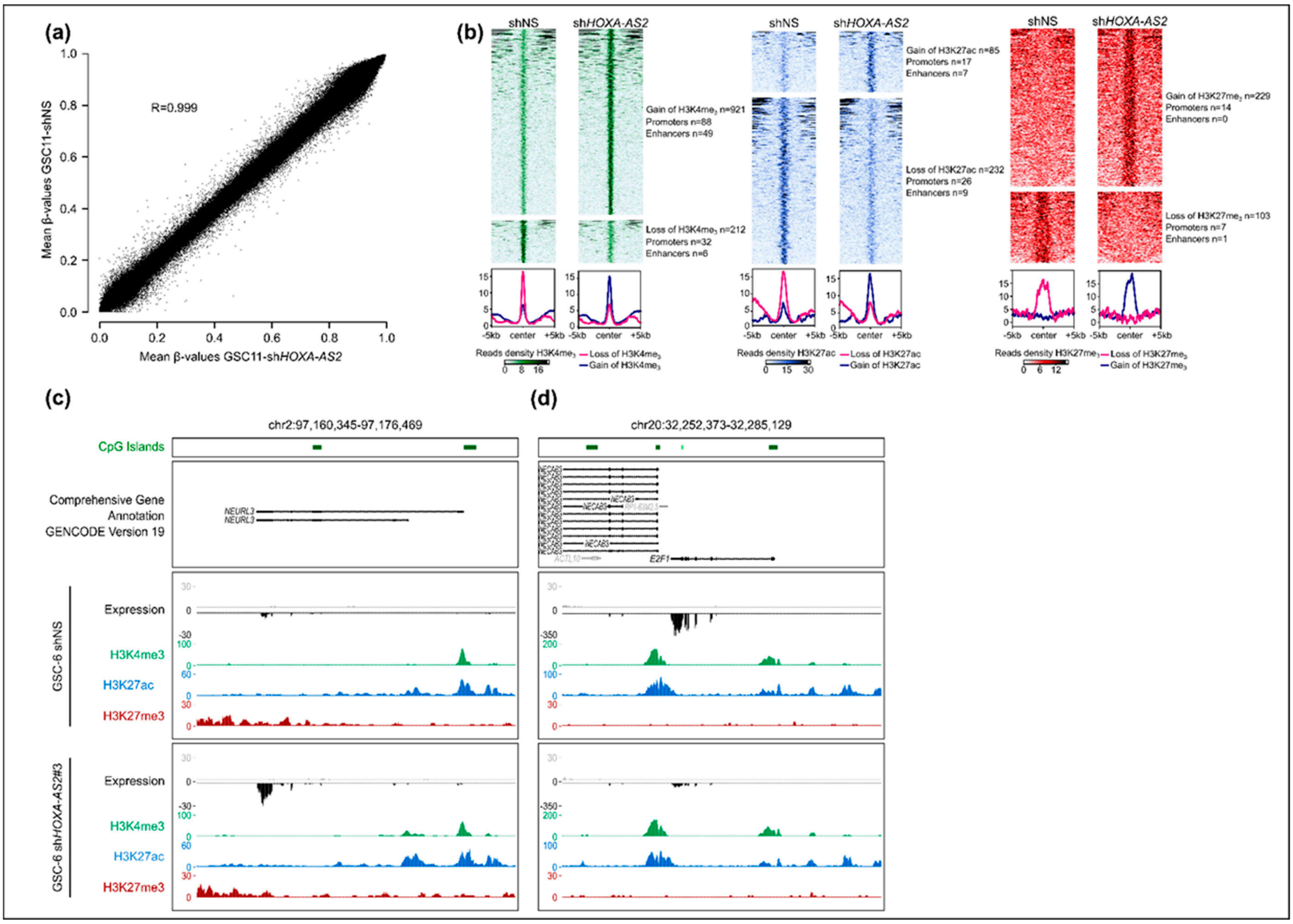

Supp figure 5: Chromatin pattern at deregulated genes following *HOXA-AS2* silencing in GSC-6 cells

(a) Scatter plots to correlate DNA methylation pattern in GSC-11 cells transduced with lentiviruses expressing shNS or *sh-HOXA-AS2)* (n=4). **(b)** ChIP-seq read density data for H3K4me3 (green), H3K27ac (blue) and H3K27me3 (red) in control (shNS) and *HOXA-AS2*-silencend GSC-6 cells. Only regions (±5 kb window) with changes (FC>2; FDR<0.01) in the level of a given mark are shown. The mean ChIP-seq signal values are shown on the lower panels. **(c) and (d)** Genome Browser view at the *NEURL3* **(c)** and *E2F1* **(d)** loci to show H3K4me3, H3K27ac, H3K27me3 enrichment, and the strand oriented RNA-seq signal in GSC-6 cells transduced with shNS or shHOXA-AS2#3.
